# Anthropometric Indicators and Dietary Intake in Toddlers Aged from 12 to 24 Months Who Attended Private Clinics in the Metropolitan Area of Guadalajara

**DOI:** 10.3390/children10071259

**Published:** 2023-07-21

**Authors:** Citlalli Álvarez-Zaragoza, Edgar M. Vásquez-Garibay, Carmen Alicia Sánchez Ramírez, Alfredo Larrosa Haro

**Affiliations:** 1 Instituto de Nutrición Humana, Hospital Civil de Guadalajara Dr. Juan I. Menchaca, Salvador Quevedo y Zubieta # 350, Col. Independencia, Guadalajara CP 44280, Mexico; citlalli.nutricion@hotmail.com (C.Á.-Z.); alfredo.larrosa@academicos.udg.mx (A.L.H.); 2 University of Colima, Avenida Universidad #333, Colonia las Víboras, Colima CP 28000, Mexico; carmen_sanchez@ucol.mx

**Keywords:** toddlers, feeding, anthropometric indicators, nutrients, sex

## Abstract

The objective was to evaluate the anthropometric indicators and dietary intake of toddlers attending private clinics in Guadalajara. In a cross-sectional study, 101 toddlers aged 12 to 24 months were included. They were born full term, had an adequate weight for gestational age, and attended private clinics in Guadalajara. Two 24 h dietary recalls were administered. Anthropometric measurements were taken, and anthropometric indices were estimated. Student’s *t* test or the Mann–Whitney U test, chi-square test, and odds ratio were used for quantitative or qualitative variables. Males had lower Z scores for the weight/age index than females. During the week, energy intake was excessive in males [OR = 5.5 (95% CI 1.4, 20.8)], and cholesterol intake was insufficient in females [OR = 3.03 (95% CI 1.2, 7.1)]. On weekends, energy [OR = 2.5 (95% CI 1.1, 5.7)] and fiber intake [OR = 3.1 (95% CI 1.2, 7.8)] were insufficient in females. Most of the toddlers who attend the private clinics in the upper-middle socioeconomic stratum of the Guadalajara Metropolitan Area had excessive protein intake, excessive consumption of added sugars was frequent, and there was insufficient intake of vitamin D and calcium. Sex was shown to be a factor influencing nutrient intake in these toddlers aged 12–24 months. Males had a Z-score of weight/age lower than females, suggesting nutritional risk.

## 1. Introduction

When toddlers aged between 12 and 24 months are integrated into the family diet, they are exposed to a wide variety of foods for the first time; therefore, this period is critical for the establishment of healthy eating practices that will have a direct effect on children’s health and nutritional status [1,2]. Hence, it is essential to ensure an adequate diet to guarantee the optimal intake of energy, macronutrients, and micronutrients [3].

In Mexico, inadequate feeding practices have been described in toddlers and older children [3], including a low prevalence of breastfeeding, low consumption of foods rich in iron, and high consumption of ultra-processed foods and sugary drinks [4]. These inadequate eating habits lead to insufficiency or deficiency of iron, zinc, calcium, vitamin D, fiber, etc., and excessive intake of energy, added sugars, saturated fats, and sodium [3]. In addition, micronutrient deficiencies in infants and toddlers have been recognized to have long-term adverse consequences for growth and development and direct consequences throughout life [5].

The assessment of nutritional status is a sensitive tool that indicates a child’s health [6]. Protein-energy malnutrition is a problem in children under five years of age worldwide. It has been estimated that there are 52 million children with wasting, 17 million with severe wasting, and 155 million with stunting [7]. On the other hand, overweight and obesity in the pediatric age group are leading to a double burden of malnutrition since 41 million children under five years of age are overweight or obese [7,8]. In Mexico, the National Health and Nutrition Survey (ENSANUT in Spanish) [9] reported a prevalence of overweight and obesity of 8.2% in children aged 0–4 years; however, 22.2% of this population was at risk of being overweight, and 14.2% were affected by short stature. Therefore, the evaluation of nutritional status in this age group through the use of anthropometric indicators is essential for the identification of problems related to malnutrition syndrome [1,10]. Therefore, the objective of the study was to evaluate the anthropometric indicators and dietary intake of toddlers aged 12–24 months who attended private clinics in the Metropolitan area of Guadalajara.

## 2. Patients and Methods

**Patients:**  

This analytical cross-sectional study included 101 healthy toddlers aged 12 to 24 months who were born full term, had an adequate weight for gestational age, and attended private clinics in the Guadalajara Metropolitan Area in the period from January 2020 to January 2021. The exclusion criteria were toddlers with genopathies, major congenital malformations, and/or chronic diseases; infants who did not present for a consultation with their mother or the person in charge of feeding them; and toddlers who attended daycare. The sample size was calculated as 89 toddlers with the following equation:

n = (Z − α + Z-β)^2^ ∗ p (1 − p)/δ^2^; n = (1.96 + 0.84)^2^ ∗ 0.5 (1 − 0.5)/(0.425 − 0.575)^2^

where α: level of significance or probability of type 1 error: Z α/2 = 0.025 (1.96); β: type 2 error probability: 0.20 = power 80% (0.84); p = 0.5 (50% of probability accepted as true); δ^2^ = 15% of the accepted distance of the true proportion. n = 89+ and 30% of exclusions, n = 115. A non-probabilistic sampling system was carried out for convenience and focused on concentration sites.

**Methods:**  

### 2.1. Techniques and Instruments for Obtaining Information

**Dietary surveys.** The same researcher administered two 24 h dietary recalls (one on weekdays and the other on weekends). The mother or person in charge of feeding the toddler was asked about all foods and drinks consumed during the previous 24 h period. The amount of each food or ingredient consumed was estimated using household measurements (including spoons, cups, slices, handfuls, etc.) and food replicates.

The consumption of human milk and its nutritional supply were estimated considering the toddler’s age in months and using the energy supply that human milk offers according to age [11,12]. The total amount of another type of human milk substitute, including cow’s milk, was considered according to the amount in milliliters reported by the parents for the previous day. For the analysis of the two dietary surveys, Nutrimind^®^ software (CDMX, Mexico) was used. For the interpretation of energy and nutrient intake, the adequate percentage was used for all reported nutrients with the exception of saturated fat and added sugars: <85% was considered insufficient; 85–115% was considered sufficient; and >115% was considered excessive [13]. For saturated fat, <10% was considered adequate and >10% was considered excessive [14], and for added sugars, <5% of the total energy value of the diet was considered adequate [15].

**Socioeconomic level survey**. The socioeconomic level was determined using the questionnaire developed by the Mexican Association of Market Intelligence and Public Opinion Agencies [16].

The information from both surveys was obtained directly through an interview with the mother or person in charge of feeding the toddler.

**Anthropometric measurements.** Weight was measured without clothing on a pediatric scale with a capacity of 20 kg and a minimum reading of 5 g (Seca 334, Hamburg, Germany). Length was measured with an infantometer (Seca 416, Hamburg, Germany). An observer held the toddler’s head with the vertical plane portion firm against the vertical part of the infantometer, and the investigator straightened the toddler’s knees and placed the feet with the toes up against the movable vertical area of the infantometer, making an angle of 90°. The mid-upper arm circumference (MUAC) of the left arm was measured with the toddler sitting on the knees of their mother or caregiver. The toddler’s arm was placed at a 90° angle, and half the distance from the acromion to the olecranon was marked as the midpoint. Subsequently, the measurement was carried out with the arm extended; the middle part of the arm was previously marked with a flexible steel metal anthropometric tape measure (Lufkin Model W606PM; Cuernavaca, México). The tricipital skinfold (TSF) was measured in the previously marked medial postero-internal part of the left arm. With the arm extended in a resting position, the adipose tissue layer was separated from the muscle, and the measurement was obtained three seconds after placing the caliper (Lange skinfold, Minneapolis, MN, USA). For subscapular skinfold (SSF) measurement, the lower edge of the scapula was located, and the fold measurement was taken horizontally at an angle of 45°. Later, the measurement was made with a plicometer (Lange skinfold, MI, USA). The measurement was obtained three seconds after the caliper was placed.

**Anthropometric indicators:** Once the anthropometric measurements of the toddlers were obtained, the data were entered into Anthroplus^®^ software (WHO, (accessed on 1 April 2022) https://www.who.int/tools/child-growth-standards/software) to obtain the Z scores of the anthropometric indicators: weight/age, length/age, weight/length, BMI/age, TSF/age, SSF/age, and MUAC/age.

### 2.2. Statistical Analysis

Descriptive statistics were obtained, and the normality of the data distribution was evaluated with the Kolmogorov–Smirnov test. If the distribution of the data was normal, Student’s *t* test was used for independent samples; otherwise, the Mann–Whitney U test was used for the contrast analysis of the means or medians for quantitative variables. Subsequently, the chi-square test was used for qualitative variables, and odds ratios were used to identify the probability of association and its epidemiological significance. A value of *p* ≤ 0.05 was considered significant. Statistical analysis was performed using the SPSS^®^ version 22 program.

## 3. Results

**General characteristics.** A total of 101 toddlers aged 12 to 24 months were included, and three of them were excluded due to incomplete surveys. Of the total sample of toddlers (*n* = 98), 48% were female and 52% were male; the average age was 16 months. Considering the socioeconomic level, 81% of the toddlers belonged to the A/B level, and 19% belonged to the C+ socioeconomic level [16]. The A/B level mostly comprises households in which the head of the family has completed professional studies (82%); ninety-eight percent of these households have internet access at home, and households in this level invest the most in education. Level C+ households have one or more vehicles, and 91% have internet access at home. Slightly less than one-third of the income is used for food (31%) [16].

**Anthropometric indicators.** In the total population, the average weight was 10.1 kg, the average length was 78.5 cm, and the average BMI was 16.3 kg/m^2^. Males had higher weight, height, and BMI values than females. The anthropometric indicators of the arm showed that males had higher MUAC and TSF values than females (Table 1). The Z scores of the anthropometric indicators in the toddlers were within normal values. According to sex, females had a lower Z score for the SSF/age indicator, and in contrast, males had a lower Z score for the weight/age indicator (Table 1).

**Dietary intake.** Table 2 shows the energy and nutrient intake on a weekday for the total population and by sex. Males had a higher intake of energy, carbohydrates, proteins, fats, cholesterol, iron, and zinc than females. Similarly, on the weekend, males had higher energy, carbohydrate, protein, fiber, and iron intakes than females (Table 3). The comparison of a day during the week vs. weekend showed that intake of energy (kcal/d; *p* = 0.003), HC (g/d; *p* = 0.045), fiber (g/d; *p* = 0.016), added sugars (kcal/d; *p* < 0.001), and iron (mg/d; *p* = 0.01) were higher on a weekday. There were no significant differences in the rest of the nutrients (data not presented in the table). When analyzing the adequate percentage of energy and nutrient intake on a weekday, it was found that most toddlers had insufficient energy intake, which was more frequent in females. In contrast, males were more likely to have excessive energy intake than females [RM= 5.5 (95% CI 1.4, 20.8) *p* = 0.003] (Table 4). Likewise, on the weekend day, most of the toddlers had insufficient energy intake, and there was a higher probability of insufficient intake in females than in males [OR = 2.5 (95% CI 1.1, 5.7) *p* = 0.03] (Table 5).

On a weekday, most of the toddlers had a sufficient intake of carbohydrates (CHO), and an excessive intake of CHO was more frequent in males than in females. When analyzing the percentage distribution of proteins, the majority of toddlers had excessive protein intake, and the frequency was similar in females and males. A relevant finding was that 31% of the toddlers had insufficient fat intake, and the frequency was similar in females and males. Most of the toddlers had insufficient cholesterol intake, which was more frequent in females than in males [OR= 3.03 (95% CI 1.2, 7.1) *p* = 0.010] (Table 4).

In relation to protein intake, similar findings were found for the intake on a weekend day and a weekday since most toddlers had an excessive intake; a similar frequency was also found for both sexes. Likewise, a similar percentage was found for insufficient fat intake, and the frequency of insufficient intake was similar in females and males. Cholesterol intake was insufficient in most toddlers, both females and males (Table 5). Likewise, most toddlers had insufficient fiber intake on both the weekday and weekend day (Table 4 and Table 5). However, on the weekend day, females were more likely to have insufficient intake than males [OR= 3.1 (95% CI 1.2, 7.8) *p* = 0.014] (Table 5).

A relevant finding was that 46% of the toddlers had an excessive intake of added sugars on the weekday, which was more frequent in females than in males (Table 4). On the weekend day, 29% of the toddlers had an excessive intake of sugars, with a similar frequency in females and males (Table 5). When analyzing the intake of micronutrients on the weekday and weekend day, it was found that most of the toddlers had insufficient iron intake, which was more frequent in females than in males. Regarding zinc intake, it was observed that approximately 40% of the toddlers had insufficient intake, and it was more frequent in females than in males. Most of the toddlers had insufficient intake of vitamin D, and it was similar in females and males. Additionally, most toddlers had insufficient calcium intake, which was similar in females and males (Table 6).

## 4. Discussion

It is known that infants and toddlers aged 6 to 24 months are in a critical stage because, during this period, their nutritional status can be compromised and can affect the child for life [17]. Therefore, it is important to monitor children at this stage of life. One of the most important tools for carrying out this surveillance is the evaluation of anthropometric indicators.

**Anthropometric indicators.** Our study showed higher weight, BMI, MUAC, and TSF values in males, attributable to biological sex differences [18]. The Z scores of the anthropometric indicators in the toddlers studied were found to be within normal values, which reflected a normal nutritional status. However, when we divided the toddlers by sex, we found that the females had a lower Z score of the SSF/age indicator, which reflected that they had a lower fat mass reserve than the males. These findings agree with other studies on malnutrition syndrome in females in Mexico and have important epidemiological and socioanthropological implications due to a potential discriminatory effect toward the female gender [19]. This could be related to the cultural perceptions of mothers and/or caregivers, since the perception that males have a greater appetite would lead to earlier complementary feeding, which could eventually cause greater adiposity in males [20].

Traditionally, it is believed that females are more frequently malnourished [21]. However, in the present study, the Z score of the weight/age indicator was lower in males, suggesting that they could have a higher nutritional risk. This finding is consistent with various studies that have shown that male sex has a greater effect on nutritional status and that males have a higher risk of malnutrition [20,22]. It has been reported that the nutritional status of preschool children, both males and females, under five years of age is associated with various factors, such as the mother’s education level, socioeconomic level, region, and place of residence [23]. Thus, the observed differences suggest a complex interplay of social, environmental, and genetic factors [20].

**Dietary intake.** The transition period of feeding from 12 to 24 months is very important because in this period, eating patterns are established that lead to eating habits for life and influence overall health [1]. To date, there is more availability of energy-dense and low-nutrient foods and beverages, which can deviate from healthy food consumption patterns among toddlers, compromising their growth, development, and nutritional status [24].

Recent studies suggest that children’s dietary intake and food preferences are influenced by gender; however, to date, there are few studies on toddlers, as most are focused on late preschoolers and schoolchildren [25]. In the present study, it was observed that in toddlers aged 12 to 24 months, the intake of energy, carbohydrates, and fats on the weekday and on the weekend day was adequate [1,13,14,26]. However, other studies have reported that toddlers have excessive energy intake and insufficient fat and carbohydrate intake [27]. Males were more likely to have excessive energy intake than females [RM = 5.5 (95% CI 1.4, 20.8), *p* = 0.003]. In relation to the ingestion of CHO, males had a tendency toward higher intake. These findings are also consistent with what has been reported by other studies in which males were observed to have a higher intake of CHO [28].

Approximately 30% of the toddlers had insufficient fat intake, with a similar frequency in females and males, and most toddlers had insufficient cholesterol intake, which was more frequent in females than males [OR = 3.03 (95% CI 1.2, 7.1), *p* = 0.010]. Insufficient fat intake has been reported in toddlers [24]. However, contrary to this finding, excessive fat intake has also been reported [29], including in Mexican toddlers [30]. Saturated fat intake was adequate [26]. In contrast to this finding, excessive saturated fat intake in infants and toddlers has been reported [24]. There were no differences between females and males in saturated fat intake. In contrast, other authors have reported that males had a higher intake of saturated fat [28]. A worrying finding was that protein intake was excessive [26], especially in males. This finding agrees with those reported by other authors [27,28]. However, other studies have shown that toddlers had adequate protein intake [24,29]. Higher protein intake increases circulating amino acid concentrations, which could increase the secretion of insulin and insulin-like growth factor 1 (IGF-1) and result in rapid weight gain and increased body fat deposition [31].

Fiber intake was insufficient [26], which could be attributable to the low consumption of fruits and vegetables in the toddlers in the present study and agrees with what was reported by Villalpando in Mexican toddlers [30]. Likewise, other authors have also described that toddlers have insufficient fiber intake [24,27,28,29]. A diet adequate in dietary fiber during this stage of life is important because it can promote healthy bowel function and decrease the risk of developing diabetes and obesity later in life [32]. On a weekend day, females were more likely to have insufficient fiber intake than males; this sex difference finding is different from that reported in other studies [28].

Vitamin D and calcium intake was insufficient in most toddlers, and the frequency was similar in females and males. Other authors have also reported that toddlers have insufficient intake of vitamin D and calcium [24,28,29,33,34]. Vitamin D is mainly produced by endogenous synthesis in the skin from exposure to sunlight, and only 10% of vitamin D comes from the diet [33]. However, toddlers and preschoolers now routinely spend most of their time indoors, whether in the home, daycare centers, or kindergarten, which could cause vitamin D deficiency. Vitamin D deficiency is a problem worldwide, and a high prevalence of deficiency of this nutrient has been reported even in Mexican children [33]. In addition, inadequate intake of vitamin D is a problem that begins early in postnatal life and is prevalent throughout life. Therefore, it is essential to ensure an adequate intake of vitamin D in children, considering that this nutrient has multiple important biological and physiological functions [29]. In Mexico, the main foods that provide vitamin D are dairy products and eggs [33,34]; therefore, the promotion of the ingestion of these foods is very important to achieve the necessary requirements, combined with sun exposure [33]. Likewise, adequate calcium intake in the diet of children at an early age is important to achieve normal skeletal growth and optimal bone mass accretion [35]. Therefore, initiatives are required to ensure adequate intake of this inorganic nutrient in this age group to achieve optimal bone health.

Regarding the ingestion of zinc, approximately 40% of the toddlers had insufficient intake of this inorganic nutrient, and it was more frequent in females than in males. However, other studies have reported adequate intake in toddlers [24,29]. Insufficient zinc intake and, consequently, zinc deficiency, is associated with slow growth, increased morbidity and mortality, and low weight gain in children under five years of age [36]. Most toddlers had insufficient iron intake, and it was more frequent in females [30]; no sex differences were found [28]. However, other authors have reported adequate iron intake in toddlers [24,29]. Iron deficiency and anemia are associated with impaired cognitive development, inadequate behavioral development, poor school performance, and decreased productivity later in life [37]. Therefore, it is important to monitor whether the iron intake requirement is met in this age group. In the present study, sodium intake was adequate; nevertheless, excessive intake of this inorganic nutrient has been reported in toddlers [24,29,30]. It has been reported that excessive sodium exposure early in postnatal life could favor the development of a salt preference that would persist into later life and could lead to increased blood pressure [38,39].

It is important to note that the consumption of added sugars on the weekday and on the weekend day was excessive, with no differences by sex. The consumption of added sugars in children under 24 months of age [40] is not recommended, and consumption is excessive even if we consider the recommendations established by the European Society of Pediatric Gastroenterology, Hepatology and Nutrition (ESPGHAN), which indicate that consumption should be avoided if the energy from sugars is exceeded by 5% [15]. This finding agrees with those reported by other authors, who have observed that in Mexican toddlers, there is a high consumption of sugary drinks and foods with added sugars [4], and it agrees with other studies of toddlers [24,29]. Excessive intake of sugars in the first two years of life could displace the intake of other essential nutrients and cause micronutrient deficiencies, obesity, and chronic noncommunicable diseases in adulthood [24]. Although the preference for sweet foods is innate and has a strong genetic component, it could be modified or reinforced by pre- and postnatal exposure [15]. Therefore, the findings observed in our study could be considered a focus of attention.

The studied population belonged to the upper-middle socioeconomic stratum and has been little explored in Mexico. Therefore, there is little information related to feeding patterns and anthropometric indicators in this population of toddlers between 12 and 24 months of age. In the present study, toddlers had a potentially risky eating pattern due to deficient and/or excessive consumption of certain nutrients.

**Limitations and strengths**. A potential limitation of our study was the use of 24 h dietary recall surveys, as there may be caregiver recall bias. However, it should be noted that nutritionists who were previously standardized and trained before data collection to reduce interviewer errors administered all 24 h dietary recall surveys. Additionally, caution must be exercised when interpreting the micronutrient data, as a 24 h survey may not accurately represent micronutrient intake. Another limitation was the sample size and the cross-sectional design of the study, which did not allow a causal effect to be established, so it is appropriate to continue exploring this area and carry out other studies with a qualitative design and longitudinal observation. Finally, another limitation is that the study did not really claim to be representative of the middle and upper-middle socioeconomic strata of the Guadalajara metropolitan area, since the sampling was not probabilistic for convenience and focused on concentration sites; therefore, the results cannot be extrapolated to the universe of the study.

The main strength was the analysis of dietary nutrient intake using two 24 h dietary recall surveys, one weekday and one weekend, in toddlers aged 12 to 24 months. Another strength was the comparison of nutrient intake by gender, since, to date, there have been few studies that have included the analysis of nutrient intake in this specific age group. Despite its limitations, this study provides new relevant information on feeding practices in toddlers aged 12 to 24 months based on a sample obtained from private clinics in the upper-middle socioeconomic stratum of the Guadalajara Metropolitan Area, Mexico.

## 5. Conclusions

Toddlers aged 12 to 24 months who attend private clinics in the upper-middle socioeconomic stratum of the Guadalajara Metropolitan Area had a normal nutritional status. Males had higher weight, BMI, MUAC, and TSF values attributable to biological sex differences and to a complex interplay of social, environmental, and genetic factors. The Z score of the weight/age indicator was lower in males, suggesting that they could be at greater nutritional risk. In the whole study group, the intake of protein and added sugars was excessive, while the intake of fiber, vitamin D, and calcium was insufficient. In addition, sex was a factor influencing nutrient intake because males had higher energy, carbohydrate, protein, and iron intake, and females had lower cholesterol and fiber intake. It would be convenient to continue exploring the feeding patterns and nutritional status in either sex of toddlers of 12 to 24 months of age from all socioeconomic strata, through studies with a longitudinal design and a representative sample of the metropolitan area of Guadalajara.

## Figures and Tables

**Table 1:** Anthropometric indicators in the total population and by sex.

Total Population (*n* = 98)				Females (*n* = 47)			Males (*n* = 51)			*p*
Indicator	X	SD	Limits (Min. to Max.)	X	SD	Limits (Min. to Max.)	X	SD	Limits (Min. to Max.)	
Weight (kg)	10.1	1.4	8.0–15.0	9.4	1.0	8.0–13.0	10.6	1.5	8.0–15.0	<0.001
Height (cm)	78.5	5.0	70–94	77.0	4.2	70–87	79.8	5.4	72–94	0.005
BMI (kg/m^2^)	16.3	1.4	13.8–19.5	15.9	1.2	13.8–18.3	16.6	1.5	13.9–19.5	0.007
MUAC (cm)	14.8	1.3	12.5–18.0	14.4	1.1	12.5–17.5	15.1	1.3	13.0–18.0	0.005
TSF (mm)	7.7	2.1	4.0–17.0	7.5	1.9	4.5–12.5	7.9	2.3	4.0–17.0	0.337
SSF (mm)	5.7	1.4	4.0–10.0	5.4	1.2	4.0–10.0	6.0	1.5	4.0–10.0	0.040
Indicators (Z score)										
Weight/age	−0.27	1.01	−4.01–2.18	−0.49	0.90	−2.13–1.33	−0.05	1.06	−2.26–2.38	0.031
Length/age	−0.52	1.27	−4.01–2.18	−0.72	1.34	−4.01–2.18	−0.33	1.17	−2.64–2.07	0.131
Weight/Length	−0.02	0.94	−2.07–2.05	−0.18	0.80	−1.56–1.55	0.12	1.05	−2.07–2.05	0.103
BMI/age	0.06	0.97	−2.17–2.01	−0.07	0.86	−1.72–1.62	0.19	1.07	−2.17–2.01	0.181
TSF/age	−0.24	1.34	−3.53–3.94	−0.37	1.27	−2.82–2.21	−0.13	1.40	−3.53–3.94	0.369
SSF/age	−0.72	1.31	−2.94–2.38	−1.04	1.20	−2.94–2.16	−0.42	1.35	−2.90–2.38	0.019
MUAC/age	−0.24	1.34	−3.53–3.94	−0.37	1.27	−2.82–2.21	−0.13	1.40	−3.53–3.94	0.369

Statistical analysis: Student’s *t* test for independent samples; BMI: body mass index; MUAC: mid-upper arm circumference; TSF: tricipital skinfold; SSF: subscapular skinfold.

**Table 2:** Intake of energy and nutrients during the week in the total population and by sex.

	Total Population (*n* = 98)		Females (*n* = 47)		Males (*n* = 51)		*p*
	Median	Percentiles	Median	Percentiles	Median	Percentiles	
		25–75		25–75		25–75	
Energy (kcal/d)	894	646–1080	833	639–953	978	711–1215	0.008
Carbohydrates (g/d)	110	89–132	103	80–124	116	93–151	0.017
Carbohydrates (%)	50	46–55	49	45–54	51	46–58	0.407
Proteins (g/d)	34	26–46	33	25–42	39	29–51	0.05
Proteins (%)	16	13–19	16	13–20	15	13–19	0.842
Fats (g/d)	34	21–43	32	20–38	40	22–48	0.040
Fats (%)	33	28–39	33	28–40	33	27–38	0.809
Saturated fats (g/d)	6	3–9	5	3–8	7	4–12	0.094
Cholesterol (mg/d)	187	53–406	125	44–279	275	58–440	0.05
Fiber (g/d)	10	7–13	9	7–12	11	7–16	0.098
Added sugar (g/d)	7	0–23	9	0–24	5	0–22	0.744
Micronutrients							
Sodium (mg/d)	347	217–537	358	229–513	331	207–559	0.856
Iron (mg/d)	6	4–11	5	4–10	7	5–12	0.006
Zinc (mg/d)	3	2–6	3	1–5	4	2–6	0.048
Vitamin D (µ/d)	1	1–3	1	1–3	1	1–3	0.354
Calcium (mg/d)	349	166–622	342	125–577	373	190–689	0.258

Statistical analysis: Mann–Whitney U test; g: grams; mg: milligrams; µg: micrograms; d: day.

**Table 3:** Energy and nutrient intake on weekends in the total population and by sex.

	Total Population (*n* = 93) ^1^		Females (*n* = 45) ^1^		Males (*n* = 48) ^1^		*p*
	Median	Percentiles	Median	Percentiles	Median	Percentiles	
		25–75		25–75		25–75	
Energy (kcal/d)	847	546–1069	681	518–1011	954	646–1134	0.019
Carbohydrates (g/d)	104	71–134	89	65–118	118	88–144	0.003
Carbohydrates (%)	50	48	48	44–53	51	47–55	0.168
Proteins (g/d)	32	25–41	28	24–37	35	29–44	0.008
Proteins (%)	16	14–20	16	14–18	16	14–20	0.725
Fats (g/d)	33	20–44	28	19–38	37	22–45	0.063
Fats (%)	34	38–35	35	29–39	32	27–38	0.288
Saturated fats (g/d)	5	3–10	4	3–8	6	3–14	0.262
Cholesterol (mg/d)	169	55–428	133	39–276	212	66–443	0.128
Fiber (g/d)	9	6–13	8	4–10	10	7–15	0.003
Added sugar (g/d)	5	0–18	5	0–19	4	0–16	0.667
Micronutrients							
Sodium (mg/d)	357	212–560	314	191–550	390	234–629	0.260
Iron (mg/d)	6	4–8	5	4–7	7	5–11	0.017
Zinc (mg/d)	3	2–5	3	1–4	3	2–5	0.096
Vitamin D (µ/d)	1	1–3	1	1–2	1	1–3	0.120
Calcium (mg/d)	377	205–536	360	203–446	391	214–612	0.185

Statistical analysis: Mann–Whitney U test; g: grams; mg: milligrams; µg: micrograms; d: day. ^1^ There were two missing data points for females and three for males on weekends.

**Table 4:** Interpretation of energy and nutrient intake using the adequate percentage for a weekday in the total population and by sex ^a^.

Variables	Total (*n* = 98)		Females (*n* = 47)		Males (*n* = 51)	
	n	%	n	%	n	%
**Energy ^1^**						
Insufficient	41	42	24	51	17	33
Sufficient	40	41	20	43	20	39
Excessive	17	17	3	6	14	28
**Carbohydrates (%) ^1^**						
Insufficient	16	16	8	17	8	16
Sufficient	62	64	32	68	30	59
Excessive	20	20	7	15	13	25
**Proteins (%) ^1^**						
Insufficient	2	2	2	4	0	0
Sufficient	29	30	14	30	15	29
Excessive	67	68	31	64	36	71
**Fats (%) ^1^**						
Insufficient	30	31	15	32	15	29
Sufficient	53	54	25	53	28	55
Excessive	15	15	7	15	8	16
**Cholesterol ^1^**						
Insufficient	60	61	35	75	25	49
Sufficient	9	9	2	4	7	14
Excessive	29	30	10	21	19	37
**Saturated fat ^2^**						
Sufficient	86	88	40	85	46	90
Excessive	12	12	7	15	5	10
**Fiber ^1^**						
Insufficient	61	62	32	68	29	57
Sufficient	22	22	12	25	10	20
Excessive	15	15	3	6	12	23
**Sugar ^3^**						
Sufficient	53	54	23	49	30	59
Excessive	45	46	24	51	21	41

**^a^** Dietary Reference Intake. Institute of Medicine of the National Academies Press, 2006. ^1^ Insufficient: <85%; sufficient: 85–115%; excessive: > 115%; ^2^ Adequate: < 10%; excessive: > 10%. ^3^ Sugars: < 5% of the total energy value of the diet. Excessive energy intake: male [OR = 5.5 (95% CI 1.4, 20.8) *p* = 0.003]; excessive carbohydrate intake: male [OR = 1.9 (95% CI 0.7, 5.4) *p* = 0.146]; insufficient cholesterol intake: females [OR = 3.03 (95% CI 1.2, 7.1) *p* = 0.008].

**Table 5:** Interpretation of energy and nutrient intake using the adequate percentage for a weekend day in the total population and by sex ^a^.

Variables	Total (*n* = 93)		Females (*n* = 45)		Males (*n* = 48)	
	n	%	n	%	n	%
**Energy ^1^**						
Insufficient	43	46	26	58	17	35
Sufficient	33	36	13	29	20	42
Excessive	17	18	6	13	11	22
**Carbohydrates (%) ^1^**						
Insufficient	12	13	6	13	6	12
Sufficient	68	73	34	76	34	71
Excessive	13	14	5	11	8	17
**Proteins (%) ^1^**						
Insufficient	2	2	0	0	2	4
Sufficient	15	16	8	18	7	15
Excessive	76	82	37	82	39	81
**Fats (%) ^1^**						
Insufficient	30	32	13	29	17	35
Sufficient	46	50	23	51	23	48
Excessive	17	18	9	20	8	17
**Cholesterol ^1^**						
Insufficient	60	65	32	71	28	60
Sufficient	8	8	3	7	4	8
Excessive	25	27	10	22	15	32
**Saturated fat ^2^**						
Sufficient	76	82	38	84	38	79
Excessive	17	18	7	16	10	21
**Fiber ^1^**						
Insufficient	63	68	36	80	27	56
Sufficient	20	21	8	18	12	25
Excessive	10	11	1	2	9	19
**Sugar ^3^**						
Sufficient	66	71	33	73	33	69
Excessive	27	29	12	27	15	31

**^a^** Dietary Reference Intake. Institute of Medicine of the National Academies Press, 2006. ^1^ Insufficient: <85%; sufficient: 85–115%, excessive: >115%; ^2^ Adequate: <10%; excessive: >10%; ^3^ Sugars: <5% of the total energy value of the diet; insufficient energy intake: females [OR = 2.5 (95% CI 1.1, 5.7) *p* = 0.03]; Insufficient fiber intake: females [OR = 3.1 (95% CI 1.2, 7.8) *p* = 0.014].

**Table 6:** Interpretation of micronutrient intake according to the adequate percentage for one day during the week and one day during the weekend in the total population and by sex ^a^.

Variables	Total (*n* = 98)		Females (*n* = 47)		Males (*n* = 51)	
	n	%	n	%	n	%
**Weekday**						
**Sodium**						
Insufficient	88	90	43	96	43	90
Sufficient	5	5	2	4	3	6
Excessive	5	5	0	0	2	4
**Iron**						
Insufficient	43	44	26	55	17	33
Sufficient	17	17	7	15	10	20
Excessive	38	39	14	30	24	47
**Zinc**						
Insufficient	39	40	23	49	16	31
Sufficient	17	17	8	17	9	18
Excessive	42	43	16	34	26	51
**Vitamin D**						
Insufficient	96	98	47	100	49	96
Sufficient	1	1	0	0	1	2
Excessive	1	1	0	0	1	2
**Calcium**						
Insufficient	71	73	36	77	35	69
Sufficient	18	18	9	19	9	17
Excessive	9	9	2	4	7	14
**Weekend (*n* = 93)**						
**Sodium**						
Insufficient	86	93	43	96	43	90
Sufficient	5	5	2	4	3	6
Excessive	2	2	0	0	2	4
**Iron**						
Insufficient	42	45	26	55	19	37
Sufficient	31	33	15	32	17	33
Excessive	20	22	6	13	15	29
**Zinc**						
Insufficient	34	37	22	47	17	33
Sufficient	21	22	9	19	8	16
Excessive	38	41	16	34	26	51
**Vitamin D**						
Insufficient	90	98	47	100	48	94
Sufficient	3	3	0	0	2	4
Excessive	0	0	0	0	1	2
**Calcium**						
Insufficient	74	80	41	87	38	74
Sufficient	13	14	5	11	8	16
Excessive	6	6	1	2	5	10

**^a^** Dietary Reference Intake. Institute of Medicine of the National Academies Press, 2006. Insufficient: <85%; sufficient: 85–115%, excessive: >115%.

## Data Availability

The databases from which the data for this manuscript were obtained can be found at the Institute of Human Nutrition of the University of Guadalajara. Email: inhu@cucs.udg.mx; vasquez.garibay@gmail.com. Phone +523336189667.
